# Professionals’ Understandings of and Attitudes to the Prevention of Sexual Abuse: An International Exploratory Study

**DOI:** 10.1177/0306624X20919706

**Published:** 2020-05-23

**Authors:** Kieran McCartan, Kasia Uzieblo, Wineke J. Smid

**Affiliations:** 1 University of the West of England, Bristol, UK; 2 Forensic Care Specialists, Utrecht, The Netherlands; 3 Ghent University, Belgium; 4 Vrije Universiteit Brussel, Belgium

**Keywords:** international, public health, sexual abuse, prevention, intervention

## Abstract

Sexual abuse is a global issue and, therefore, responding to and preventing sexual abuse are global challenges. Although we have examples of and evidence for sexual abuse prevention initiatives internationally, these tend to come from a small, select group of countries (i.e., United Kingdom, United States, Canada, Germany, Netherlands, New Zealand, and Australia) and not from a broader global pool. This article will present the qualitative data from an online study (*n* = 82), covering 17 countries, on professionals’ (i.e., people working in the arena of sexual offending from a clinical, criminal justice, policy, research, and/or practice perspective) perceptions sexual abuse prevention in theory, practice, and policy. The article identifies three main themes: (a) professionals’ understandings of the prevention of sexual abuse, (b) public understanding of sexual abuse prevention, and (c) governmental attitudes towards, and support of, sexual abuse prevention programs. The article highlights that, although there are similar understandings of sexual abuse prevention internationally, practice is characterised by national differences in the funding of, provision of, and public/policy perceptions of prevention as well as its impact on offending.

In the last few decades, there has been a growing recognition of the depth and extent of sexual violence globally (National Sexual Harm Resource Centre, 2015; United Nations Children’s Fund [UNICEF], 2014). This increased global sociopolitical recognition of sexual abuse corresponds to several interrelated factors, including increased investment in sexual violence education, increased reporting of historical cases, and a growing recognition that anyone can be a victim of sexual abuse which is, in part, seen as a consequence of the #METOO movement. In the last decade, in particular, we have seen a rise in the reporting of sexual abuse, current and historical, linked to institutions, sports clubs, charities, college campuses, and the church (Australian Human Rights Commission, 2017; Tabachnick et al., 2016; Vertommen et al., 2018), and an increased media profile of sexual violence (Harper & Hogue, 2017). However, sexual violence reporting and conviction rates vary widely between and within countries, dependent on the size, culture, and economic status of the country which makes determining a global prevalence rate difficult (UNICEF, 2014; World Health Organization [WHO], 2014; see special edition of *Sex Offender Treatment*, McCartan, 2018, for a broader international perspective). Nevertheless, research indicates that 13% of children worldwide are victims of sexual abuse and that prevalence is higher for girls (18%) compared with boys (8%) (Stoltenborgh et al., 2011) and that abuse is more likely to be committed by a partner (30%) as opposed to a nonpartner (7%) (WHO, 2014). The consequences of sexual abuse are the same internationally, including physical, mental, reproductive, and sexual health lifelong impacts (Felitti & Anda, 2009; Vertommen et al., 2018), but victim’s experiences of access to services and ability to recovery can vary within and between countries. One of the challenges in reporting, recording, and responding to sexual violence, or abuse, is often times difficult to define as it can mean a range of behaviours and thought patterns which may or may not be illegal, which may or may not include a contact offence or a knowing victim and which may or may not be considered a psychological, social, or interpersonal issue. Despite knowing of these international variations there has been limited international and transnational comparative research relating to the prevention of and responses to sexual violence (Krahé, 2018; Krahé et al., 2014; Ma, 2018; Stoltenborgh et al., 2011), which is something that the field needs to address given the relevant interconnection of research, practice, and policy. For the purposes of this article we are considering sexual violence to mean any behaviour or action that results in a criminal offence, and therefore the prevention of sexual violence or abuse is anything that would stop a sexual offence from (re-)occurring.

Historically, most of the published research on the perpetration of sexual abuse relates to the management of people convicted of a sexual offence, their treatment, rehabilitation, punishment, and community management with reducing recidivism being the main driver (see Laws & O’Donoghue, 2016 for a broader discussion). Research and practice, therefore, focuses on relapse prevention and not preventing first time offending. However, over the last 20 years, since seminal work in the early 2000s (Laws, 2000; Smallbone et al., 2008; Wortley & Smallbone, 2006), the research and practitioner community have started to advocate for a public health approach to preventing sexual abuse resulting in a slow but steady cultural shift. This movement is based upon research and practice, that demonstrates that sexual abuse is a life course and multi-disciplinary issue impacting not only individuals, but communities and society (please see, Brown, 2017; Fenton et al., 2016; McKibbin & Humphreys, 2020; Nelson, 2016; Rudolph & Zimmer-Gembeck, 2016; Tabachnick et al., 2016, for a broader discussion of the sexual abuse prevention literature and the use of public health models in responding to sexual abuse). The public health approach offers a unique insight into preventing and responding to sexual abuse by focusing on the safety and benefits for the largest group of people possible and providing a comprehensive response to the problem (Kemshall & Moulden, 2016; Laws, 2000; McCartan et al., 2015; Smallbone et al., 2008; Tabachnick, 2013; Wortley & Smallbone, 2006). A public health approach allows drawing on multi-disciplinary knowledge and perspectives offered by medicine, epidemiology, sociology, psychology, criminology, education, and economics, among others. Therefore, a public health approach enables both macro (societal, community, and institution based) and microlevel (individual, family, and relational) solutions to population level problems, which sexual abuse is. Sexual abuse is a clear example of a public health issue as it affects all levels of society, occurs internationally, and has a multitude of impacts upon the individual, community, and society. Therefore, aligning sexual abuse prevention to Epidemiological Criminology (EpiCrim), which is “the study of anything that affects the health of a society, be it: crime, flu epidemics, global warming, human trafficking, substance abuse, terrorism or HIV/AIDS” (Lainer, 2010). The use of EpiCrim to understand sexual abuse is not new (Skvortsova, 2013) but it is important as it allows a preventive approach to be taken, rather than simply focusing on a reactive (i.e., after the fact) and purely putative criminal justice approach (McCartan et al., 2015); this means that we can be innovative in many more ways to tackle sexual abuse.

A core component of the EpiCrim approach is a drive towards shared language, vision, and meanings for professionals (Lainer, 2010) which is happening in sexual abuse prevention with many professional organisations (for instance, Association for the Treatment of Sexual Abuse [ATSA], National Organisation for the Treatment of Abuse [NOTA], the Dutch chapter of the Association for the Treatment of Sexual Abuse—Netherlands [NL-ATSA]), and academic journals (Willis & Letourneau, 2018) taking on a preventive, person first, health-based approach.

When discussing sexual abuse as a public health issue we tend to focus on three levels of prevention (primary, secondary, and tertiary) (Brown, 2017); but, there is also a fourth level of prevention (quaternary) (Jamoulle, 2012) which is not discussed as much (see Table 1).

**Table 1.** The Four Stages of Sexual Abuse Prevention.

Type of prevention	Definition (health)	Definition (criminal justice)	Definition (sexual abuse)	Examples
Primary	Action to prevent disease in people who feel well	Action to prevent criminogenic and/or risky behaviour in people who are not criminogenic and/or risky.	Raise public awareness of the reality of sexual abuse and dispel common myths about victims and preparators. Which enables individuals and communities to be better at identifying sexual abuse, risky behaviours and be better able to support people impacted by sexual abuse. Increased education leads to increased awareness and more proactive behaviour.	Public education campaigns, bystander intervention, eradicating child sexual abuse, and so on.
Secondary	Action to detect disease at an early state in people who feel well	Action to detect criminogenic and/or risky behaviour at an early stage in people without a criminal conviction	Enabling “at risk” populations to understand their potential risks, triggers and the potential outcomes of them. This means that they can seek appropriate support and be empowered to seek help. Individuals and communities better understand risk and therefore are better able to help people manage their own (potential) risk.	Project Prevention Dunkelfeld, Stop SO, Safer Living Foundation, Lucy Faithful, Help Wanted! Stop It Now!, The Global Prevention Project, and so on.
Tertiary	Action to reduce symptoms and complications of disease in people who feel sick	Actions, treatments and/or interventions to reduce criminogenic and/or risky behaviour in people with a conviction	Working with people convicted of sexual offences to hold them accountability for their past problematic behaviour, get support and move forward, integrate back into their communities. These interventions move people towards an offence-free lifestyle and encourage desistence. They help people manage their own risk (i.e., treatment programs and interventions).	Treatment programs and interventions for people who have committed sexual abuse and so on.
Quaternary		Action taken to protect individuals with a criminal conviction from criminal justice interventions that would result in future criminogenic and/or risky behaviour	This enables people to successfully integrate back into the community by protecting people from collateral consequences or risk management policies and practices. This is done through supportive integration programs that help the person who has sexually abused, aid their re-entry and support them proactively to negative the range of policies and practices that negate their integration.	Probation, MAPPA, Circles Of Support, and Accountability.

*Note.* MAPPA = Multi-Agency Public Protection Arrangements.

Regarding sexual violence prevention, the core aim of these four levels is to prevent offending, protect the public, reduce the impact of sexual violence and manage risk (McCartan et al., 2015; Smallbone et al., 2008). These preventive stages work within a socioecological framework that targets the individual-, the interrelationship-, the community- and the societal-level (Brown, 2017; Krug et al., 2002; Shields & Feder, 2016). However, international research in sexual abuse prevention indicates that most interventions happen at the primary (e.g., interventions for people with sexual interests in children but who have not committed any offence) and tertiary levels (e.g., treatment programs and interventions for people convicted of a sexual offence), with a growing, but limited, body of interventions at the secondary level (e.g., Eradicating Child Sexual Abuse [ECSA], 2020; Safer Living Foundation, 2020; Stop it Now!, 2020; Troubled Desire, 2020) and quaternary levels (e.g., Circles of Support and Accountability). In the field of sexual abuse, quite often tertiary and quaternary prevention are seen as the same type of prevention intervention, that is, relapse prevention, in criminal justice terms either treatment and/or punishment. But quaternary prevention is more than that as it is also about harm reduction, desistence, and community integration (see Table 1). Quaternary prevention is about reducing the negative impact of overcriminalization on men convicted of a sexual offence so that success is not hampered by confounding variables or unwanted, unnecessary factors. Most professionals and policy makers do not talk about quaternary prevention in respect to people who have committed sexual offences even though it is happening daily in practice (see Table 1). The vast majority of research in the prevention of sexual abuse comes from most of Westernised, anglophone countries (i.e., Australia, United Kingdom, Canada, America, and Canada); therefore, raising the question of whether the range and type of preventive interventions varies country by country, where best practice occurs, and how to translate best practice globally? In addition, research on the prevention of sexual abuse and public health models tends to focus on its prevalence rates and piloting small scale interventions, but not on the way professionals, policy makers and society (i.e., media and public) think about prevention. It is important to recognise broad based, and specific, understanding of sexual abuse prevention as these understanding can shape the form that the implementations of policy and practice have, as well as their success in the real world.

The aim of this research is to understand professional (i.e., professionally trained people who work with individuals convicted of a sexual offence) attitudes to and understandings of the prevention of sexual abuse. The study’s objective is to clarify professional attitudes to see whether there is commonality across them as to what prevention is and what it means in practice, as many professional practices are now prevention informed with an assumption of a shred understanding. Given that sexual abuse prevention started as a Westernised, anglophone concept, an additional aim is to explore how prevention is translated into other countries and cultures. This is important as we need to know what professionals, working in forensic mental health, the community and policy, think in respect to prevention so that we can guarantee that the interrelationship between research, policy, and practice is fit for purpose. Consequentially, this research is to our knowledge the first international qualitative study to examine professionals’ attitudes to the prevention of sexual abuse and the reality of it in the real world.

## Method

### Design

The current article focuses on professional attitudes to the prevention of sexual abuse internationally. As the research wanted to understand participants attitudes to the prevention of sexual abuse it was decided that a qualitative methodology was the best approach to take as it allowed participants to write what they wanted free of researcher bias, and as qualitative research is generally seen as a good methodology for emerging and/or conflicted research areas (Robson & McCartan, 2016).The research is part of a larger study looking at professional attitudes to the assessment, management, treatment, and integration of people who have sexually offended. The study is a mixed methods online questionnaire with 68, English language open and closed questions, which took 30 min to complete, that was built on and hosted through Qualtrics software.

### Sampling and Participants

The survey sampled members of an international organisation, with 3,000+ members across 20 countries, involved in assessment, treatment, and integration back into the community of people who have been convicted of a sexual offence. The researchers sought, and obtained, approval from said organisation to sample its members and did so via social media, direct email, and their related Listservs as well as mailing lists. The survey had 82 respondents, of those 46 were female (57%) and 36 were male (43%). The participants spanned 17 countries with a mixed response rate, for some countries had many participants (i.e., Australia, New Zealand, and Italy) and other had few (Denmark, Ireland, Israel, Japan, Puerto Rico, Singapore, Sweden, and Switzerland) (see Table 2).

**Table 2.** Participants Role/Career by Country or Residence.

Role/career	Number of participants	Country of employment
Police	1	New Zealand
Probation officer	2	Australia, Netherlands
Prison staff	3	Belgium, Canada, United Kingdom
Social worker	1	Canada
Therapist/Treatment provider	43	Australia, Belgium, Canada, Germany, Ireland, Israel, Italy, Netherlands, New Zealand, Puerto Rico, Sweden, Switzerland, United Kingdom, United States
Researcher/academic	13	Canada, Denmark, Germany, Italy, Japan, United States
Policy maker/Government	4	Canada, Netherlands, Singapore, United States
Third sector organisation	2	Italy, United States
Other	6	Australia, United Kingdom, United States
Missing data	7	

### Materials

The online survey was developed by several researchers, including but not limited to the authors,^1^ focusing on the main issues in the field of sexual abuse which included the prevention, assessment, and management of people convicted of a sexual offence. In addition, the survey was sent to members of the research organisations and ethics committee for input. The survey was developed with a robust rigour that ensured validity and reliability. The ad hoc developed online survey consisted of 68 different types of questions (i.e., closed-ended, open-ended, and Likert-type scale questions) with four questions directly related to the prevention of sexual abuse. The open-ended questions, which this article is based upon, used free text boxes which allowed the participant to write as much as they liked and consisted of the following:

1. Please describe what you think the term “sexual abuse prevention” means?
2. Do you think that colleagues and professionals who work in the field of sexual abuse, in the same country as you, understand what is meant by the prevention of sexual abuse?
3. Do you think that members of the public, in the same country as you, understand what is meant by the prevention of sexual abuse?
4. Does your country of employment have any sexual abuse prevention programs? If so could you please name them?

Some wrote in their mother tongue which was then translated into English before analysis.

### Ethics

The research was approved by the University of the West of England, Bristol ethics committee and adhered to the British Society of Criminology’s and all the partner agencies, where they had one, codes of ethical research conduct, and good research practice. The ethical issues that the research had to consider included participant anonymity, data sharing, data storage, informed consent and confidentiality, which were highlighted in the consent form that participants completed. Having clear ethical guidelines and procedures were particularly salient, given the international nature of the research.

### Analysis

The qualitative data were analysed via thematic analysis (Braun & Clarke, 2006) by the first author, with a number of themes emerging from the reading and coding phases of the analysis. The initial analysis identified seven themes linked to the four research questions, two of these themes where particularly small, only having a couple of identifiable quotes in each, including (a) defining sexual abuse prevention; (b) types of sexual abuse prevention; (c) public understandings of sexual abuse prevention; (d) media understandings of sexual abuse prevention; (e) national policy responses to sexual abuse; (f) international policy responses to sexual abuse prevention; and (g) the finical? implications of sexual abuse prevention. The second and third authors then reviewed the analysis and the three authors discussed the seven themes agreeing that they could be condensed into three larger ones, which where (a) understanding and explaining the prevention of sexual abuse (defining sexual abuse prevention; types of sexual abuse prevention); (b) public understanding of sexual abuse prevention (public understandings of sexual abuse prevention; media understandings of sexual abuse prevention); and (c) governmental attitudes towards, and support of sexual abuse prevention programs (National policy responses to sexual abuse; International policy responses to sexual abuse prevention; Finical implications of sexual abuse prevention). Qualitative data were subsequently framed in relation to the overall research aims and objectives which forms the basis of the analysis presented below.

## Results

### Understanding and Explaining the Prevention of Sexual Abuse

Participants across all 17 countries tended to agree on what the prevention of sexual abuse meant

Preventing sexual abuse from ever happening, but also preventing it will happen again if it already happened. (Belgium therapist/treatment provider)

However, some of the participants provided a more nuanced understanding of prevention indicating that it could refer to preventing first time offending as well as relapse prevention, recidivism, related to known offenders

As applied to non-offenders, the prevention of sexual offenses, by understanding laws related to the illicit/illegal use of the internet, risky (potentially illegal) sexual behaviours and identifying psychologically troubled, isolated adolescents, who have a history of being sexually abused, for treatment. Applied to recidivism of those already registered as sex offenders, sex abuse prevention must be tailored to the nature of the offense or crime and the individual’s psychological needs. Without addressing the aetiology of sexual offense there is strong likelihood substantial treatment never occurred and abuse may reoccur. (U.S. therapist/treatment provider)

Reduction of the criminogenic needs of the offenders, and promoting an effective social reintegration of the person into the community. (Italian researcher/academic)

Interestingly, one participant believed that sexual abuse prevention was much broader than sexual abuse and should include sexual harassment as well

Early intervention with problem sexual behaviour and trauma in children; safety and consent empowerment education in early learning centres and primary and secondary schools, quality assessment and treatment of young people exhibiting HSB, assessing and treating adults who abuse, anti-harassment training in the workplace, treatment for all offenders, online help and anonymity to those wanting help for online behaviour, understanding of sexual abuse dynamics for all teachers and social workers and police and nurses, etc. (New Zealand therapist/treatment provider)

A cross section of the participants (i.e., 60 participants, from 13 countries; 72%) directly linked sexual abuse prevention to public health approaches, stating that there were many levels/stages of prevention and that each one was different; which is not surprising given the nature of these debates at conferences, in international journals and that these participants came from Westernised countries. Interestingly, one participant identified quaternary prevention, whereas many professionals, especially nontreatment providers, only discussed primary and secondary prevention

I think we have to differentiate between primary (avoid occurrence of sexual abusive behaviour), secondary (helping at-risk persons), tertiary (avoiding recidivism), and quaternary (avoiding unnecessary and excessive treatment) prevention. (German researcher/academic)

That is a very broad question; there are so many levels to prevention, primary, intermediate and secondary. Most of the focus in NZ is on primary prevention, with a shift to secondary prevention. (New Zealand police participant)

Building upon this, a large proportion of the participants, that is, 80 participants (90%) across all 16 countries, thought that prevention was not just about the individuals but about communities and society as well

Engaging in whole of community interventions that reduce factors that often co-exist with sexual abuse (including social attitudes towards woman and children, ideas around encouraging speaking up and seeking help, bystander education, services for people before they have acted on abusive or unhelpful sexual thoughts). (Australian therapist/treatment provider)

Informing public in a correct way about what sexual abuse is and what are the consequences for the victims, but also informing correctly about sex offenders and the possibility of treatment and management. Offering services to those at risk of committing offenses and preventing relapse with evidence-based programs. (Italian therapist/treatment provider)

Working together as a community to reduce the incidence and severity of sexual abuse. (U.K. therapist/treatment provider)

This led the participants to state that prevention activities were wide, multi-faceted and linked to several different populations in different ways.

Working with people who are vulnerable to becoming sexual abusers to prevent that from happening. (Canadian therapist/treatment provider)

Information for caregivers and potential victims (children) about different forms of abuse, how to react and where to find help, in the ideal case help for individuals with deviant sexual fantasies or ideas before any illegal act happens, adequate treatment and necessary surveillance for offenders who are at risk of re-offending (in line with the good lives-model). (German therapist/treatment provider)

Educating the public, proper policing, rehabilitation of offenders. (Israeli therapist/treatment provider)

Info work on keeping kids and their parents informed about private parts, body rules and the legal rules around sex behaviour. Good sexual education in school. (Swedish therapist/treatment provider)

It means putting in place a comprehensive set of guidelines, policies, education from which develops a culture in which sexual abuse is less likely. (New Zealand therapist/treatment provider)

#### Summary

The participants’ understandings and attitudes reflect existing research and training on the prevention of sexual abuse (Brown, 2017; McCartan et al., 2015; Shields & Feder, 2016). The qualitative data suggest that there is not a great deal of variance in participants’ attitudes to the prevention of sexual abuse based upon country of employment or professional role; but we cannot say this definitively given the small and opportunistic sample that we have (McCartan, 2018). The participants all recognise that the prevention of sexual abuse is a public health issue and that by preventing sexual abuse we could alleviate a range of negative outcomes for victims, perpetrators, both of their networks and society. In addition, the participants recognise that, while there are a range of different approaches to prevention, education is the most central one, believing that this should be delivered through the development of bespoke, fit for purpose programs/interventions for different audiences (i.e., at risk populations, parents, adolescences, communities) (Colin-Vezina et al., 2013). Interestingly participants see the prevention of sexual abuse as a broad-based community issue and not just the responsibility of professionals. The majority of the participants who commented, worked in the same field, mainly as a therapist/treatment provider who are actively engaged in the international research and treatment community (i.e., attending ATSA, The Australian and New Zealand Association for the Treatment of Sexual Abuse [ANZATSA], NOTA, and related conferences), and therefore may have been exposed to the same training, education and resources. Therefore, we have to make sure that we are not overstating professional understandings when in reality the majority of comments come from one target group. This raises the issue of a potential echo chamber impacting professional views of the prevention of sexual abuse. Does the fact that there is a limited number of researchers, trainers and conferences linked to sexual abuse prevention mean that we are getting the same story, or at least variants of it, all the time? Which is relevant as it means that as the field of prevention, treatment and management of people convicted of a sexual offence may move forward based on research, but that practice may follow at a distance. Not having national, bespoke approaches for implementation of prevention research and practice is challenging as the bulk of prevention research comes from certain countries and may not be culturally, or socially, translatable at a practical level to all countries globally (Tabachnick et al., 2016); although, this is starting to change with more international studies taking place (Abeid et al., 2014; Kamimura et al., 2016).

### Public Understanding of Sexual Abuse Prevention

All the professionals believed that the public attitudes to people who have been convicted of a sexual offence impacted their opinions on the feasibility of prevention interventions and whether they would work in practice with preventive measures

They (the public) would focus on prevention of existing/identified offender from reoffending rather than prevention of those who are at risk or offending or changing rape culture. (Canadian researcher/academic)

They (the public) would not understand the holistic nature of the approach that is required due to their level of fear and misinformation about sexual offenders and sexual abuse risk. (Belgium therapist/treatment provider)

The public would simplify the issue and advocate for prevention to be incarceration i.e. if you’re concerned about someone lock them up because the damage they may potentially inflict is harsher than the restriction on their freedom. (U.K. therapist/treatment provider)

#### Summary

In sum, the participants did not believe that the public fully understood what the prevention of sexual abuse entailed. The prevention of sexual abuse, especially secondary prevention, is a relatively new research, political and societal discourse and, therefore, compared with other parts of the sexual abuse arena we do not have a strong evidence base to determine public opinion. The limited evidence base tends to focus on public attitudes to primary (Kemshall & Moulden, 2016) and tertiary prevention (Centre for Sex Offender management, 2010; Höing et al., 2016), with a growing body of work linked to secondary (Tabachnick et al., 2016) as well as quaternary prevention (Richards & McCartan, 2017). Professional opinions of public attitudes towards sexual abuse prevention seem to be based upon their implicit, common sense theories and experiences rather than evidence based explicit theories. The findings from this research do indicate similar professional concerns internationally (i.e., negative attitudes, lack of understanding, etc.), which suggests an opportunity for professionals to come together with the public as well as with policymakers to create a dialog and develop resources around sexual abuse prevention. The participants believed that public understanding of sexual abuse prevention, to respond to the crisis of sexual abuse, is impacted by existing, limited and fearful perceptions of sexual abuse (Harper et al., 2017; Jahnke, 2018). Therefore, to enable the public to better understand prevention, there needs to be more education and increased societal debates around the reality and benefits of prevention (Harper et al., 2017). Currently, sexual abuse prevention is mainly discussed in professional circles, with limited media and press coverage in some countries (McCartan, 2018), which means that the professional, policy and public discourses are not well joined up impacting the feasibility of implementing prevention initiatives and there resulting take up by the community.

### Governmental Attitudes Towards, and Support of, Sexual Abuse Prevention Programs

The majority of participants, overall and across all countries sampled, believed that their governments approach to sexual abuse was punitive (45 participants, 55%), with less thinking that their governments were rehabilitative (31 participants, 37%) or preventive (18 participants, 22%). Only 16 participants (20%) believed that their governments provided enough, or any, financial resources to support the prevention of sexual abuse, with many participants stating that their respective governments did not promote prevention as much as they could

They (the government and policymakers) focus primarily on the treatment of victims and not on the sex offenders. (Puerto Rican therapist/treatment provider)

Can’t think of any, we did have a program on safe relationships in schools but the government withdrew funding following a scare campaign it was encouraging homosexuality. (Belgium, other)

We had recent revisions to the sex educational program in schools—this caused significant protest from parents. Parents fail to understand the importance of education of children in the prevention of abuse. (Canadian therapist/treatment provider)

#### Summary

Although the participants believed that sexual abuse prevention was not well funded or a government priority, they did indicate that the majority of the countries involved in the study had sexual abuse prevention programs/interventions in place (see Table 2). This suggests that in some form sexual abuse prevention exists internationally, although as previously stated, mainly primary and tertiary prevention with secondary and quaternary emerging over the last few years. The latter may however be more of a grass roots movement as opposed to a strategic government one. The prevention programs that exist internationally seem to be based in a small number of high-profile countries that lead the research and evidence base agenda in prevention (Australia, United Kingdom, Canada, United States, New Zealand, and Germany) as well as in a few smaller, more liberal countries (Sweden, Denmark, and Italy) (McCartan, 2018). The countries that have a number of established prevention programs are those that provide the main sexual abuse research and training, so it is not a surprise that they have progressed prevention initiatives more coherently. The participants indicated that the prevention projects, if they were able to identify them, were not always easy to access locally, regionally, nationally or internationally. Participants demonstrated that, while there was willingness to engage in sexual abuse prevention in policy and practice, it was not always a priority for organisations; there was a lack of local, national and international resources and/or initiatives across all four prevention stages. Therefore, while prevention exists internationally it doesn’t seem to be as well established as it needs to be. Interestingly, professional knowledge of prevention programs was not always reflective of reality of actual programs, for instance, the Belgian participants failed to mention existing programs in their country, which is surprising as both Stop it now! and CoSA do exist in Belgium.

## Conclusion

The current study has offered the largest and most coherent international snapshot of professional’s attitudes to, understandings of and experiences of the sexual abuse so far. The paper indicates that there is a growing recognition of what the prevention of sexual abuse is among professionals and that this is broadly consistent with most professionals internationally. However, we need to keep in mind that there might be an echo chamber effect as most participants sampled are probably exposed to the same research, training, and publications, given their affiliation with the leading research and practice organisations in the field (i.e., ATSA, NOTA, International Association for the Treatment of Sexual Offenders [IATSO], ANZATSA, NL-ATSA, etc.).

Although the professionals felt that other people working in the field of sexual abuse understood prevention, which again maybe a confirmation bias, and why it was important they did not feel that this translated to the public or government/policy makers understanding or attitudes. The participants felt that the public and the governments in the countries that they worked had a poor understanding of what prevention of sexual abuse was and why it mattered. The participants believed that these misconceptions and poor understandings where linked to the complexity of the sexual abuse, myths surrounding perpetrators, and prevailing attitudes to what responses to sexual abuse should be, which is reflected in that the participants believed that sexual abuse was seen as a mainly criminal justice, reactive issue. Although, not all the participants believed that the public and/or policy makers had a poor understanding of sexual abuse prevention across the board, but rather they believed that there was still a distance to go before sexual abuse prevention was a social norm and central to policy and practice (McCartan, 2018). The research reiterated that sexual abuse prevention is mainly thought of as being at the primary, secondary and tertiary levels, with most professionals not recognising quaternary prevention. Where quaternary prevention does exist, it maybe be confused or tied together with tertiary prevention, like Circles of Support and Accountability which is actually quaternary prevention but often seen as tertiary prevention. Professionals may need to do some work to better clarify this distinction. Several of the countries sampled had some prevention interventions already in place, albeit not across all four levels of the prevention spectrum. But these did not seem to be widely dispersed even not among professionals. In addition, a lot of these countries thought that their governments were not pro-prevention. The professionals argued that these programs need to have a stronger evidence base, a clearer rationale and a better multi-disciplinary, comprehensive network.

This study has limitations, including, the fact that it was in English, and therefore only English speaking or bilingual speakers responded to the questionnaire, which meant that it was a self-selecting sample, and consequentially it had a relatively small sample size. Notwithstanding, it is the largest international studies of its kind, as existing empirical research on understandings of sexual abuse prevention tend to be in country, small scale and intervention focused, rather than, like with this study, a piece of research that aims to explore professionals practical and everyday views of sexual abuse prevention. If the research was to be replicated it would be expanded from its initial form to having a broader remit, to sample a broader international participant pool and to be an independent study; however, to do this we need to determine best practice in overcoming language and narrative barriers. Although the survey is small scale, it does identify that the understandings of the prevention of sexual abuse and its importance are spread throughout the international professional community and across several anglophone and nonanglophone countries. But there seem to be major international differences being in respect to access to programs, resources, and governmental support (McCartan, 2018). The paper indicates that we need to continue developing good practice and sharing ideas to make prevention more internationally accessible and consistent, which would result in a broader international evidence base and a reduction in the potential echo chamber effect in professional understandings. Professional bodies, like the ones sampled in this study, have a role to play in promoting research, training and policy linked to the prevention of sexual abuse within as well as across countries.

## Notes

1 The survey was developed in conjunction with the ATSA international committee.
